# Thinking Style Moderates the Impact of the Classroom Environment on Language Creativity

**DOI:** 10.3390/jintelligence12010005

**Published:** 2024-01-08

**Authors:** Suqin Lin, Wenjin Duan, Yifan Wang, Haijun Duan

**Affiliations:** 1 School of Preschool Education, Xi’an University, Xi’an 710065, China; linsqedu@xawl.edu.cn; 2 Key Laboratory of Modern Teaching Technology, Ministry of Education, Shaanxi Normal University, Xi’an 710062, China; wangyifan0929@snnu.edu.cn

**Keywords:** thinking style, classroom environment, Chinese language creativity

## Abstract

The classroom environment significantly affects the development of creativity. This study examined the impact of the classroom environment on students’ creativity and the moderating role of thinking styles in this relationship. For this study, we recruited 451 students from six secondary schools. Data were collected using the Chinese Language Creativity Test, Classroom Environment Inventory, and Thinking Styles Inventory. Hierarchical regression analysis examined the moderating effect of thinking styles on the relationship between the classroom environment and creativity. The results showed that peer relationships in the classroom environment negatively influence students’ fluency and originality in creativity. At the same time, teachers’ evaluation and teaching methods positively affect the fluency of creativity. Thinking styles moderated the impact of the classroom environment on language creativity. This study identified four different moderating effects: the thinking styles matching the classroom environment can enhance language creativity, whereas the mismatched ones hinder it. However, matching would limit language creativity for individuals with creative thinking styles (e.g., legislative and anarchic thinking styles), while a mismatch can boost creative performance. The findings help educators understand students’ creativity with different thinking styles in various classroom environments and provide individualized and effective strategies for optimizing educational environments and enhancing language creativity.

## 1. Introduction

The psychological study of creativity is essential to human progress (Kaufman and Sternberg 2019). Creativity has been considered one of the top four learning skills that should be cultivated among young learners in the 21st century. Meanwhile, creativity research focuses on shifting from domain generality to domain specificity. Language creativity refers to the output of original and valuable work in learning through the input of language information, the application of knowledge, problem-solving, the investigation of phenomena, and aesthetic creation (Hu et al. 2006). Language creativity exemplifies a frequently used form of creative expression in daily life, and it is widely regarded as the most salient example of everyday creativity. Moreover, studies have shown empirically that it is important for developing general thinking skills (Smogorzewska 2014). There is little research on the students’ language creativity. Exploring the factors that affect language creativity is crucial because it offers useful information on improving language creativity in educational settings. Consequently, this study adopted Chinese as a language carrier to better explore students’ language creativity. It focused on language creativity to investigate the influence of the classroom environment on language creativity and how thinking styles interact with the classroom environment to affect language creativity.

### 1.1. Language Creativity and Classroom Environment

The 4P theory of creativity simplifies the creative process into four key components: Person, Process, Product, and Press. The “Press” component highlights how the environment and external factors shape creativity. According to ecological systems theory (Bronfenbrenner 1977), classroom environments are microsystems where adolescents constantly interact with others and conduct their daily activities. The bidirectional interactions between adolescents and the classroom environments influence adolescents’ development (Kim 2015). The classroom environment is an organic combination of material, social, and psychological factors that affect the teaching process and quality. A positive classroom environment fosters the growth of divergent thinking in students by creating a conducive atmosphere for learning and expression (Hong and Song 2020). Previous studies have developed specific research on several dimensions of the classroom environment. For instance, the positive teacher-student relationship creates a safe and trusting space where students feel encouraged to express their ideas and take risks (Lodson and Ogbeba 2020). The physical environment also plays a role in nurturing students’ creativity. Studies have shown that classrooms with good acoustical quality facilitate concentration and engagement, allowing students to generate innovative ideas (Dul 2019; George and Youssef 2012). Additionally, an environment that promotes independence, risk-taking, and intrinsic motivation stimulates students’ creative thinking (Zhang et al. 2021; Richardson and Mishra 2018). Research has also focused on other specific dimensions of classroom environments that influence creativity, such as peer interactions (Wang and Murota 2016), teaching methods (Dai 2010, 2012, 2015; Smogorzewska 2012, 2014; Rojas-Drummond et al. 2008), and teacher attitudes (Newton and Beverton 2012).

These studies provide insightful information, but they fall short of considering the complex external environment because they focus on just one aspect of the classroom environment rather than considering it holistically. Rudasill et al. (2017) proposed the Systems View of School Climate (SVSC) by deconstructing prior models and empirical research on school climate and defined school climate as the affective and cognitive perceptions regarding social interactions, relationships, values, and beliefs held by students, teachers, administrators, and staff within a school. Therefore, according to the SVSC and prior literature, this study identified seven key dimensions of the classroom environment: teacher-student relationship, peer relationship, teaching methods, teacher attitude, classroom atmosphere, teacher evaluation, and physical environment. These dimensions collectively may shape the different classroom environments, directly influencing students’ creativity and development. Furthermore, fewer studies focus on the relationship between the classroom environment and language creativity. The present study fills these gaps by conducting a seven-dimensional synthesis analysis of the classroom environment and investigating the effects of each dimension on students’ language creativity. Therefore, this study proposes Hypothesis 1:

**H1:**  *Quality classroom environments, which include democratic teacher-student relationships, harmonious peer relationships, open and innovative teaching methods, tolerated and friendly teachers’ attitudes, an open-minded classroom atmosphere, positive and encouraging teacher evaluations, and a creatively friendly physical environment, provide students with an ideal environment in which to stimulate creative thinking and expression.*

### 1.2. Thinking Style, Classroom Environment, and Language Creativity

Creativity results from a mix of individual psychological mechanisms and environmental factors. Researchers have found that creativity is most effectively fostered when the external environment is constructed properly and individual differences are considered (Liu 2020). Thinking styles, referring to individuals’ preferred approaches to utilizing their abilities, encompass cognitive processes such as perception, judgment, decision-making, and problem-solving, shaping how individuals engage with intellectual tasks (Sternberg 2000; Sternberg and Grigorenko 1997). Previous studies have demonstrated that the thinking style can significantly influence some key cognitive processes involved in creativity (Eon Duval et al. 2022; Duan et al. 2020; Wang et al. 2019; Piaw 2014; Abdi 2012).

Sternberg (1988) identified 13 thinking styles based on the theory of mental self-government, which Zhang (2002) later reconceptualized into three types. Type 1 thinking styles, which tend to produce more creativity, are characterized by higher levels of cognitive complexity, including legislative (creativity), judicial (evaluation of others or products), hierarchical (task prioritization), global (holistic focus), and liberal (innovative approaches) thinking styles. Type 2 thinking style is represented by a preference for rules and lower cognitive complexity, and it includes executive (task implementation based on orders), local (detail-focused), monarchic (focusing on one task at a time), and conservative (relying on traditional approaches) styles. Depending on the needs of the task, Type 3 styles, which include anarchic (managing a variety of activities), oligarchic (working on numerous tasks concurrently without regard to priority), internal (individual work), and external (collaboration), exhibit traits of both Type 1 and Type 2 styles (Kuan and Zhang 2022).

Hongdizi et al. (2023) emphasized the need for more empirical research regarding the relationship between thinking style and creativity. Sternberg and Grigorenko (1997) pointed out that thinking style is influenced by an individual’s interactions with their environment and can be shaped and developed over time. This highlights the dynamic nature of thinking styles and their potential responsiveness to different environments. Previous research has underscored that a student’s academic achievement can be optimized when environmental factors, such as teaching strategies, align with the student’s thinking style (Ahmady et al. 2019; Chen et al. 2011; Tulbure 2011; Zhang 2004). Furthermore, it has been observed that students with varying thinking styles exhibit distinct preferences for teaching methods, underscoring the significance of recognizing individual diversity, particularly in thinking styles, within the classroom environment. Chen et al. (2011) extended this perspective by showing that when teaching strategies align with students’ thinking styles, it results in higher levels of reflection and improved academic performance.

Recognizing the influence of the classroom environment on creativity and the significance of the alignment between thinking styles and external conditions, this study addresses an important gap in the existing literature. While prior research has established the connection between thinking styles, teaching strategies, and academic outcomes, more attention needs to be paid to the specific impact of this interaction on language creativity. This interplay between the classroom environment and thinking styles is crucial for understanding language creativity development. Research has indicated that the classroom environment plays a pivotal role in shaping creativity, and previous literature suggests that individuals experience more significant growth when their thinking styles harmonize with their external environment. Therefore, our study hypothesizes that when the classroom environment aligns with students’ thinking styles, it can significantly enhance the development of language creativity. Hence, this study proposes Hypothesis 2:

**H2:**  *When students’ thinking styles match the classroom environment, language creativity will be enhanced. Creativity will be hampered if the classroom environment and the students’ thinking styles do not match.*

**Figure 1:**
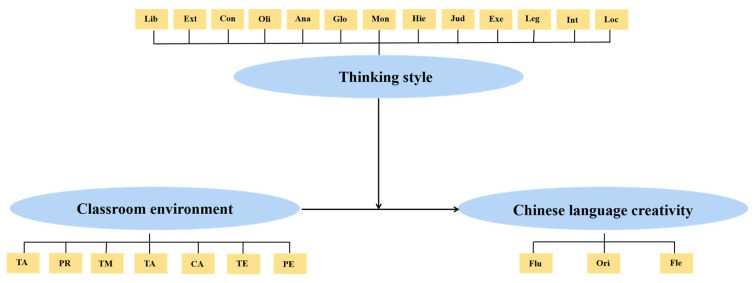
The hypothesized model. TR: teacher-student relationship; PR: peer relationship; TM: teaching methods TA: teachers’ attitude; CA: classroom atmosphere; TE: teachers’ evaluation; PE: physical environment. Flu: fluency; Ori: original; Fle: flexibility. Lib: liberal thinking style, Ext: external thinking style, Con: conservative thinking style, Oli: oligarchic thinking style, Ana: anarchic thinking style, Glo: global thinking style, Mon: monarchic thinking style, Hie: hierarchical thinking style; Jud: judicial thinking style; Exe: executive thinking style; Leg: legislative thinking style; Int: internal thinking style; Loc: local thinking style.

In summary, this study aimed to examine the association between the classroom environment and students’ language creativity and explore the impact of the interaction between the classroom environment and students’ thinking styles on their language creativity. Considering the significant role played by thinking style and the classroom environment in shaping students’ language creativity, we hypothesize that a positive match between the classroom environment and a student’s thinking style will boost their language creativity (see Figure 1).

## 2. Materials and Methods

### 2.1. Participants

Using the random sampling method, six junior high schools spanning city, county, and township levels within Shanxi Province were chosen. A total of 451 second-year students were randomly selected to complete the questionnaires. The participants comprised 198 (43.9%) males and 253 (56.1%) females. They were aged between 12 and 17. This study was approved by the Ethics Committee of the Shaanxi Normal University of China. Since the sample was large, the experimenters explained this study before performing the formal procedure. All participants provided written informed consent before participation.

### 2.2. Measures

#### 2.2.1. Classroom Environment Inventory (CEI)

Drawing upon the seven dimensions of the classroom environment identified by Rudasill et al. (2017) and the existing categorization of the classroom environment in previous studies, we conducted interviews with teachers, experts, and students to formulate an initial questionnaire for a comprehensive assessment of the classroom environment. In the end, the Classroom Environment Inventory was developed, which consists of 41 items categorized into seven dimensions: Teacher-student relationship (TR), Peer relationship (PR), Teaching methods (TM), Teachers’ attitude (TA), Classroom atmosphere (CA), Teachers’ evaluation (TE), and Physical environment (PE).

Participants rated each item on a five-point scale, ranging from 1 (completely mismatched) to 5 (completely matched). Then, a pretest was conducted, followed by analyses of reliability and validity. The Cronbach’s α of the complete questionnaire was 0.89, and for each dimension, it ranged from 0.5 to 0.7. The half-spilled coefficient for each dimension was between 0.6 and 0.7, all acceptable based on the basic requirements of psychometrics. Additionally, a confirmatory factor analysis supported the validity of the questionnaire’s final version (Table 1 presents the coefficients of the CFA model).

#### 2.2.2. Thinking Styles Inventory (TSI)

The TSI (Sternberg and Grigorenko 1997) is a self-report questionnaire in which students express their preferences. There are 13 types of thinking styles, which are classified based on function (legislative, executive, and judicial styles), form (monarchic, oligarchic, hierarchical, and anarchic styles), level (global and local styles), scope (internal and external styles), and leaning (liberal and conservative styles). Some examples of the items are “I like tasks that allow me to do things my way” (legislative), “I like situations in which it is clear what role I must play or how I should participate” (executive), and “I like to evaluate and compare different points of view on the issues that interest me” (judicial). Table 2 describes each thinking style. This study adopted a brief version of the TSI that was revised by Zhao (2006) for teenage students. It has 58 items in addition to the 104 items in the original questionnaire. A 7-point Likert scale was used in this questionnaire (1 = strongly disagree, 7 = strongly agree). Cronbach’s α for this questionnaire in this study ranged from 0.50 to 0.84. Additionally, confirmatory factor analysis was conducted to assess the construct validity of the questionnaire, and the results demonstrated a good model fit.

#### 2.2.3. Chinese Language Creativity Test

Chinese language creativity was measured using the Chinese Language Creativity Test (Hu et al. 2006). This test consisted of four parts, each with a single open-ended question that called for a particular approach and level of language knowledge. More creative thinking skills and creative imagination are essential as well. The test is suitable for individual and group administration and takes 90 min. Part I and Part II, Problem Finding and Imagination of Outcomes, are designed to measure creative language problem formulation ability and adolescents’ language imagination ability, mainly in terms of creative fluency, flexibility, and originality; Part III, Essay Writing, which focuses on aesthetic creativity of language, in terms of flexibility and originality; and Part IV, Phenomenal Exploration, which is designed to measure adolescents’ linguistic phenomenal inquiry ability, in terms of flexibility and originality.

Fluency, flexibility, and originality were rated based on the following guidelines: The number of valid answers determined fluency. The number of answer categories determined flexibility, and originality was determined based on the proportion of students who provided the same answers. Thus, answers with a similarity percentage below 5% received a score of 2, those with a similarity percentage between 5% and 10% received a score of 1, and those with a similarity percentage higher than 10% received a score of 0.

To revise the original test, a preliminary study was conducted with 256 participants randomly selected from two junior high schools in Linfen, China, including 99 males. Two postgraduate students who majored in psychology independently graded all the answers, and the inter-rater consistency was 0.85 (for each dimension, it ranged from 0.64 to 0.72). Therefore, the average scores assigned by the raters for each item were used. The internal consistency of the test was found to be 0.78 (for each dimension, it ranged from 0.69 to 0.78).

### 2.3. Procedure

**Figure 2:**
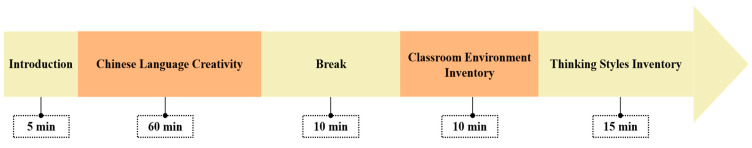
Test procedures.

The official tests were conducted in the following manner: First, we provided a brief explanation of this research intentions and requirements. Then, the participants took the Chinese Language Creativity Test, which lasted approximately 60 min. After this, there was a 10 min break. Then, the participants took the CEI, which lasted approximately 10 min. Finally, the participants took the TSI, which lasted approximately 15 min. Figure 2 illustrates this process.

As the experimenters, postgraduate students who had majored in psychology conducted the tests. They did not know the experiment design and had not been trained before taking the formal tests.

Once all three tests were finished, the experimenters collected all this papers, encoded all the booklets, and checked the answers based on the scoring principle of each test. The researchers who designed this study performed further statistical analyses.

### 2.4. Data Analysis

Hierarchical regression analysis was used to determine the moderating effect of students’ thinking style on the relationship between the classroom environment and students’ Chinese language creativity. The averaged data on classroom environment and thinking styles were standardized to avoid multicollinearity (Wen et al. 2005). Seven independent variables representing the classroom environment were entered into the first step of the regression equation, and the 13 moderating variables denoting students’ thinking styles were entered in the second step. The interaction terms (independent variable × moderating variable) were entered in the third step. Chinese language creativity had three dimensions, and each dimension was used as a dependent variable. Tolerance and variance inflation factors were used to check for multicollinearity. Tolerance ranged between 0.13 and 0.705 (>0.1), and the variance inflation factor ranged between 1.419 and 7.665 (<10); both were within a reasonable range.

## 3. Results

### 3.1. Descriptive Statistics

Table 3 presents significant differences among different level schools in the dimensions of classroom environment, particularly in the dimensions of peer relationships (*F*_(2,421)_ = 2.23, *p* < 0.05), teacher attitudes (*F*_(2,421)_ = 5.58, *p* < 0.05), and teacher evaluations (*F*_(2,421)_ = 3.06, *p* < 0.05). Teachers in city schools exhibit more positive and democratic attitudes toward students and provide relatively relaxed atmospheres. Significant differences are also observed in teacher-student relationships (*F*_(2,421)_ = 7.00, *p* < 0.001), teaching methods (*F*_(2,421)_ = 7.02, *p* < 0.001), classroom atmosphere (F_(2,421)_ = 11.12, *p* < 0.001), and physical environment (*F*_(2,421)_ = 7.47, *p* < 0.001). City schools create more open and dynamic classroom environments and generally have better facilities than rural-level schools. The moderation analysis was not performed independently for various school types to achieve a more complete distribution of the independent variable (classroom environment). Furthermore, to avoid the influence of demographic variables and other factors, such as the characteristics of the school, on this study results, they were analyzed as control variables in the subsequent analysis.

Table 4 presents the means and standard deviations of the main variables studied. Subsequently, based on students’ Chinese language creativity test scores, participants were categorized into the high language creativity group (upper 27th percentile of the distribution) and the low language creativity group (lower 27th percentile of the distribution). Table 5 presents the comparative analysis of thinking style scores between the high and low language creativity groups. It was found that the high language creativity group showed a tendency towards legislative, executive, judicial, liberal, hierarchical, anarchic, and local thinking styles. In contrast, the low-language creativity group tended to prefer monarchic, oligarchic, global, internal, external, and conservative thinking styles. The findings of earlier studies, which found that people with particular thinking styles are more creative, are primarily supported by our results (Zhang 2002). Moreover, significant differences were found in the hierarchical (*t* = 2.11, *p* < 0.05), anarchic (*t* = 2.10, *p* < 0.05), and local thinking styles (*t* = 2.07, *p* < 0.05) between the two groups. Additionally, significant differences were found in legislative (*t* = 2.60, *p* < 0.05), executive (*t* = 2.62, *p* < 0.05), judicial (*t* = 2.58, *p* < 0.01), and liberal thinking styles (*t* = 2.62, *p* < 0.01) between the high and low creativity groups.

### 3.2. The Moderating Role of Thinking Style in the Influence of the Classroom Environment on Language Creativity

Table 6 shows that when the interaction terms were entered into the regression equation, the change in *R*^2^ was 0.288, *p* < 0.001 for fluency and 0.248, *p* < 0.05 for originality. These findings verify the moderating effect of students’ thinking styles on the classroom environment’s impact on language creativity. However, for flexibility, the coefficients reveal that the moderation effect of thinking style is not significant. Therefore, in subsequent results presentations, the data with flexibility as the dependent variable will not be shown.

### 3.3. Classroom Environment and Thinking Style Effects on Fluency and Originality

Table 7 shows the impact of the classroom environment and thinking style on fluency and originality. Regarding the impact of the classroom environment, peer relationships negatively influence fluency (*β* = −0.5, *p* < 0.05) and originality (*β* = −0.188, *p* < 0.01). Meanwhile, teachers’ evaluation positively affects fluency (*β* = 0.653, *p* < 0.001). Teaching methods positively affect originality (*β* = 0.132, *p* < 0.05). Regarding the effect of students’ thinking styles, only anarchic and external thinking styles influence fluency, but their effects are opposite. The anarchic thinking style positively influences fluency (*β* = 0.409, *p* < 0.05), whereas the external thinking style has a negative impact (*β* = −0.587, *p* < 0.01).

### 3.4. The Moderating Role of Thinking Style in the Influence of Classroom Environment on Fluency

Regarding the impact of the interaction of the classroom environment and students’ thinking styles, the influence of teachers’ evaluation, teachers’ attitude, peer relationships, teacher-student relationships, physical environment, and teaching methods on students’ language creativity is highly related to students’ thinking styles.

Table 8 shows the coefficients of the moderated regressions for fluency (only significant items are presented). Teachers’ attitudes toward fluency are moderated by legislative, anarchic, and internal thinking styles (*β* = −0.605, *p* < 0.05; *β* = −0.701, *p* < 0.05; *β* = −0.978, *p* < 0.01). Anarchic thinking style also moderates the influence of teaching methods on fluency (*β* = 0.703, *p* < 0.01). Additionally, the classroom atmosphere’s impact on fluency is moderated by executive, judicial, and anarchic thinking styles (*β* = 0.623, *p* < 0.05; *β* = −0.916, *p* < 0.01; *β* = 0.700, *p* < 0.05). Peer relationships’ effects on fluency are moderated by judicial and internal thinking styles (*β* = 0.769, *p* < 0.01; *β* = −0.528, *p* < 0.05), while teaching evaluation of fluency is moderated by hierarchical and local thinking styles (*β* = 0.190, *p* < 0.01; *β* = 0.160, *p* < 0.05). Moreover, the teacher-student relationship on fluency is moderated by local thinking style (*β* = −0.568, *p* < 0.05), and global thinking style moderates the impact of the physical environment on fluency (*β* = −0.565, *p* < 0.05).

### 3.5. The Moderating Role of Thinking Style in the Influence of Classroom Environment on Originality

Table 9 shows the coefficients of the moderated regressions for originality (only significant items are presented). Regarding originality, the executive thinking style moderates the impact of teachers’ attitudes (*β* = 0.195, *p* < 0.05). The internal thinking style also moderates the influence of classroom atmosphere (*β* = 0.151, *p* < 0.05), while peer relationships are influenced by monarchic, hierarchical, and local thinking styles (*β* = −0.169, *p* < 0.05; *β* = −0.150, *p* < 0.05; *β* = 0.189, *p* < 0.05). Additionally, the teaching evaluation’s effects on originality are moderated by anarchic and internal thinking styles (*β* = −0.668, *p* < 0.05; *β* = −0.553, *p* < 0.05), and the teacher-student relationship’s effects on originality are influenced by internal thinking style (*β* = 0.193, *p* < 0.05). Lastly, the external thinking styles moderate the impact of the physical environment on originality (*β* = −0.197, *p* < 0.05).

Mainly, students’ executive, judicial, and anarchic thinking styles moderate how the classroom environment affects students’ fluency, whereas the internal thinking style moderates the relationship between the classroom environment and the originality of language creativity. The main findings of this study will be discussed in detail in subsequent sections.

## 4. Discussion

In order to increase ecological validity and explanatory power, this study examined the effects of external and internal factors, such as thinking style and the classroom environment, on language creativity. A key issue considered was the role of students’ thinking styles in the relationship between the classroom environment and language creativity. Our results showed that thinking styles significantly moderate the effect of the classroom environment on fluency and originality. However, thinking styles did not moderate the effect of the classroom environment on flexibility.

It is important to note that traditional creativity tests usually follow three dimensions: the characteristics of creative products, which emphasize the originality, uniqueness, applicability, or value of the created objects; secondly, the features of creative thinking, which emphasize the originality, fluency, and flexibility of thought processes; and thirdly, the cognitive processes involved in problem-solving. In contrast, language creativity tests assess individuals based on purpose and task, engaging with language input through listening, reading, and observation. This involves processing language information using four modes: language knowledge application, language problem formulation, and resolution, exploration of linguistic phenomena, and linguistic aesthetic creation. Ultimately, these tests evaluate individuals’ abilities to produce novel, unique, and valuable linguistic products through speaking or writing. Specifically, the primary focus of language creativity tests is testing the originality, flexibility, and fluency within individuals’ capacities in problem formulation, reading comprehension, imagination, writing conceptualization, language aesthetic appreciation, and exploration of linguistic phenomena.

### 4.1. Influence of the Classroom Environment on Language Creativity

Results revealed that teachers’ evaluation positively influenced students’ fluency, and teaching methods positively impacted students’ originality, which supports Hypothesis 1. However, peer relationships were found to have a negative effect on both students’ fluency and originality. The other dimensions of the classroom environment did not significantly impact any dimension of creativity. These findings refute Hypothesis 1.

This study reveals the different roles that teaching methods and teachers’ evaluation play in fostering the development of students’ language creativity. Teaching methods are the strategies teachers employ to assist students in acquiring knowledge, enhancing their abilities, and developing effective learning approaches to achieve the teaching objectives in this study. Positive teaching methods are characterized by openness, adaptability, and innovation. Through the adoption of open-ended teaching methods, students are encouraged to analyze issues from diverse perspectives and explore novel methodologies (Daher and Hashash 2022). Teachers can also empower students with independent learning and problem-solving skills by promoting effective learning methods, which are crucial in fostering creativity (Eshet and Margaliot 2022). Overall, our findings suggest that the pivotal role of instructional methods in enhancing the originality of students’ language creativity is attributed to teachers who utilize inspiring teaching materials, organize activities that stimulate creativity, and provide opportunities for students to engage in autonomous exploration. This fosters students’ ability for independent thought, enables them to express themselves linguistically in more creative ways, and promotes audacious language creativity. Consequently, it aids them in achieving higher scores in the originality dimension of language creativity.

Concurrently, teachers’ evaluation is defined as teachers’ verbal and nonverbal evaluations of students during their teaching activities. The potency of teacher evaluations lies in their positivity and motivational nature. Positive teacher evaluation boosts students’ confidence and encourages them to participate in class and think creatively. Specifically, teachers’ evaluation of enhancing the fluency of students’ language creativity lies in the prompt feedback provided during regular instructional activities. Through timely evaluations, teachers encourage students to offer more relevant and meaningful language expressions in their responses, enabling them to generate diverse linguistic content swiftly and flexibly. Therefore, teachers’ evaluation emphasizes stimulating students’ quantitative language creativity performance, elevating their fluency scores. Davies et al. (2013) systematically reviewed the literature on creative learning environments published between 2005 and 2011. They found that several key characteristics of the environment and conditions effectively promote the development of creative skills among children and young people. Consistent with our results, they showed that new and dynamic activities or tasks, regarded as teaching methods, drive students’ creativity. Moreover, Lodson and Ogbeba (2020) conducted a qualitative study to investigate what kind of classroom environment fosters students’ creativity. They found several critical aspects that establish a creative environment. These results correspond to the positive effect of teachers’ evaluation and teaching methods that this study shows.

Despite previous studies finding that peer collaboration is a key aspect of high-quality peer relationships closely related to students’ creative performance (Park et al. 2023), this study revealed a negative impact of peer relationships on the originality and fluency of language creativity. Considering China’s collectivist culture and the psychological development characteristics of adolescents, peer expectations and a sense of collective belonging may constrain students (Helgeson et al. 2009). Those who feel different from their peers may be reluctant to engage in behaviors that distinguish them from the majority when confronted with creative tasks. Furthermore, when peer interactions become too frequent and intense, students may lack sufficient time for personal reflection and exploration (Li and Wang 2022), limiting their ability to develop unique perspectives and express creativity.

Despite previous studies finding that peer collaboration is a crucial aspect of high-quality peer relationships closely related to students’ creative performance (Park et al. 2023), this study revealed a negative impact of peer relationships on the originality and fluency of language creativity. Considering China’s collectivist culture and the psychological development characteristics of adolescents, peer expectations and a sense of collective belonging may constrain students (Helgeson et al. 2009). Those who feel different from their peers may be reluctant to engage in behaviors that distinguish them from the majority when confronted with creative tasks. Furthermore, when peer interactions become too frequent and intense, students may lack sufficient time for personal reflection and exploration (Li and Wang 2022), limiting their ability to develop unique perspectives and express creativity.

This study has found that teachers’ attitudes, teacher-student relationships, and classroom atmosphere do not significantly impact students’ language creativity. One possible reason is the respect and reverence students have for their teachers, which are essential values in Chinese education (Dong et al. 2021). This authoritative culture may influence the interaction between teachers and students, resulting in a high level of consistency in teacher-student relationships and teachers’ attitudes, thus limiting their impact on students’ language creativity. Regarding the physical environment, different from previous research, this study mainly focused on spatial factors or other‘real’ physical factors, such as the acoustic environment and lighting system. Previous studies have shown that classrooms with good acoustical quality facilitate concentration and engagement, allowing students to generate innovative ideas (Dul 2019; George and Youssef 2012). However, our study found that the physical environment does not significantly impact students’ language creativity. This suggests that students in higher grades are more likely to be proactive in handling various physical factors, instructional facilities, and temporal and spatial environments in the classroom, thereby reducing the negative effects of unfavorable physical environments on language creativity. In future research, exploring the impact of the classroom’s physical environment on individuals from additional dimensions would be beneficial.

### 4.2. Influence of the Interaction between the Classroom Environment and Thinking Styles

This study showed that different thinking styles moderated the impact of different dimensions of the classroom environment on fluency and originality in language creativity. To some extent, this result verified our Hypothesis 2 that students’ creativity, at least fluency and originality, is enhanced when the classroom environment matches students’ thinking styles.

The TSI, based on Sternberg’s theory of mental self-government, is employed to measure the thinking styles of the participants (Chen 2022). Thinking styles from different dimensions cannot be compared with each other, whereas those from the same dimension are distinctive and comparable. However, this does not imply that individuals can be strictly categorized into any specific thinking style. On the contrary, each person exhibits varying degrees of all thinking styles. The divergence lies in the intensity of their preferences and the specific tasks and contexts that elicit such preferences. (Hammad and Awed 2022). Therefore, a high preference for one style can influence one’s behavior. For this reason, we focused on understanding the behavior patterns associated with the highly preferred thinking styles in each dimension.

Multiple moderating effects are found in this study because there are thirteen dimensions for the moderating variable (thinking styles) and seven dimensions for the independent variable (classroom environment). Traditionally, the creative learning environment had several traits, such as teachers respecting students, teachers’ tolerance for different ideas, the de-emphasis on standard answers, encouragement of original ideas, students respecting and collaborating with each other, and the flexible use of physical materials (Lodson and Ogbeba 2020). The CEI is mainly based on these traits. In this study, unlike the traditional conclusions, a fundamental hypothesis was that students’ creativity is enhanced only when the classroom environment matches their thinking style. Table 10 further illustrates the performance of a high-quality classroom environment in each dimension and the implications of having a strong preference for a particular thinking style. We also demonstrated the effect on students’ creativity when a dimension of the classroom environment matches or does not match their highly preferred thinking styles based on the results from Table 8 and Table 9. It provides four different types of moderation to help readers better understand them: matched and enhanced creativity, did not match and inhibited creativity, matched and inhibited creativity, and did not match and enhance creativity.

The first two categories aligned with Hypothesis 2. Specifically, matching hierarchical thinking style and teachers’ evaluation significantly enhances students’ originality. This can be attributed to the inherent nature of individuals who possess a hierarchical thinking style. These students are naturally inclined to prioritize tasks and methodically assess them (Zhang 2004). When this propensity aligns with teachers’ positive evaluation, it creates an environment where students feel empowered to explore unconventional ideas without apprehension. Instances of mismatch between specific thinking styles and corresponding dimensions of the classroom environment have been linked to inhibitions in creative expression. When the judicial thinking style is at odds with the prevailing classroom atmosphere, it can lead to a clash between students’ natural inclination to evaluate rigorously and an environment that thrives on open exploration. This misalignment may impede their fluency in brainstorming innovative concepts. Moreover, misalignments are also evident in internal thinking styles and their interactions with external factors. An internal thinking style that does not harmonize with the teacher’s attitude or peer relationships can hinder fluency. Additionally, the lack of alignment between the monarchic thinking style and peer relationships, as well as the hierarchical thinking style and peer relationships, has been shown to suppress students’ originality.

The other two categories deviated from the results of previous studies. Unexpectedly, when the legislative thinking style was aligned with teachers’ attitudes, as well as when the anarchic thinking style coincided with teachers’ attitudes, it negatively impacted fluency. At the same time, the mismatch between anarchic thinking style and classroom atmosphere, along with teaching methods, fosters a notable increase in fluency. Anarchic thinking styles can generate more creativity according to the needs of the task, while high levels of cognitive complexity characterize legislative thinking styles and tend to produce more creativity (Zhang 2002). Therefore, these unexpected results may be because when students have thinking styles conducive to creativity (such as legislative and anarchic), they need guidance and norms from the outside environment more. More open classroom atmospheres, teachers’ attitudes, and teaching methods cause students who think in anarchist and legislative styles to become disoriented and unable to accurately orient their thinking without adequate guidance. Although we have given reasons for these unexpected results, we acknowledge that more research is needed to confirm the accuracy of these unexpected results. Furthermore, the alignment of external thinking styles with the physical environment emerged as a factor inhibiting originality. This outcome may be attributed to the potential cognitive dissonance between the external thinking style, which focuses on collaborative ventures with others, and the physical environment, which emphasizes a creative-friendly physical setup. The latter may not provide the optimal conditions for collaboratively generating novel ideas. These results highlight the importance of educators understanding students’ thinking styles and adjusting classroom environments accordingly to enhance language creativity. This individualized approach to education can help students reach their full potential and achieve better results in the learning process.

Additionally, our results showed several mismatches in thinking style and corresponding classroom environment dimensions contributed to enhanced fluency and originality. The unmatched executive thinking style with classroom atmosphere, the unmatched judicial thinking style with peer relationships, and the unmatched internal thinking style with teachers’ evaluation all correlated with heightened fluency. These findings suggest that incongruities in these scenarios motivate students to think more flexibly and explore different perspectives, enhancing their ability to generate a wide range of ideas. Furthermore, the unmatched executive thinking style and teachers’ attitude, the unmatched internal thinking style and classroom atmosphere, and the unmatched internal thinking style and teacher–student relationship were found to promote originality. These findings show how a lack of harmony between cognitive preferences and the educational environment can encourage students to think divergently and engage in creative problem-solving, ultimately enhancing their originality. On the one hand, another possible explanation for the unmatched–enhanced results is that the environment forced the students to move beyond their comfort zones and engage with different stakeholders to come up with innovative solutions to challenges as they arose (Inayat et al. 2023). On the other hand, in those circumstances, students were compelled to think and act differently, which later enabled them to reflect on their thoughts and actions (Van Gelderen 2023). These experiences likely boosted their creativity. This notion is further supported by historical examples, such as Van Gogh, who struggled with his circumstances but still managed to create valuable masterpieces. We must stress once more that the findings of our research demand additional replication and validation investigations. In summary, the results of this study indicate to educators that, under certain circumstances, a comfortable life may fail to stimulate students’ creativity.

The moderation of the local thinking style was stronger than that of the global thinking style, mainly inhibiting students’ creativity. Since creativity involves creating new things from scratch, creators must be familiar with the whole picture of something. Then, it is easier for them to see the inner links, reconstruct, and produce new ideas. The global thinking style moderated the impact of only the physical environment on fluency. Among students with a strong preference for the global thinking style, those situated in a traditionally ‘good’ physical environment—offering ample creative space; flexible settings; and superior facilities—experienced a decline in fluency. Such students tend to adopt a theoretical approach, emphasizing the overall situation and abstract concepts when confronting problems. However, within a creative environment, striking a balance between theoretical and practical experimentation becomes crucial to avoid restricting the fluency of their creativity.

Based on these results, it seems that what we considered a “creative” classroom environment (one that has a high score for each dimension) was not always good for students’ creativity, even when their thinking style matched the classroom environment. Our assumption that the match between thinking style and classroom environment drives students’ creativity was only partially verified. A “not creative” classroom environment (one that has a low score for each dimension) can sometimes boost students’ creativity. Therefore, no golden rule exists for building a creativity-boosting classroom environment in teaching practice. Our findings call for an individualized approach to educational environment design that takes into account the intricate interaction between thinking styles and the classroom environment. This was the most valuable finding of this study.

## Figures and Tables

**Table 1:** Confirmative Factor Analysis of the Classroom Environment Inventory.

Dimensions	*df*	*χ* ^2^	GFI	AGFI	TLI	CFI	RMSEA
TR	46	232.15	0.99	0.95	0.98	0.96	0.06
PR	49	246.95	0.98	0.91	0.90	0.97	0.07
TM	38	192	0.98	0.94	0.95	0.97	0.05
TA	27	137	0.97	0.91	0.95	0.98	0.04
CA	35	178	0.96	0.96	0.95	0.95	0.03
TE	24	124	0.98	0.98	0.96	0.98	0.06
PE	47	235.6	0.99	0.95	0.96	0.96	0.05

Note: TR: teacher-student relationship; PR: peer relationship; TM: teaching methods; TA: teachers’ attitude; CA: classroom atmosphere; TE: teachers’ evaluation; PE: physical environment.

**Table 2:** Thinking styles in the theory of mental self-government (Zhang 2004).

Dimension	Thinking Style	Key Characteristics
Function	Legislative	One prefers to work on tasks that require creative strategies One prefers to choose one’s own activities
	Executive	One prefers to work on tasks with clear instructions and structures One prefers to implement tasks with established guidelines
	Judicial	One prefers to work on tasks that allow for one’s evaluation One prefers to evaluate and judge the performance of other people
Form	Hierarchical	One prefers to allocate attention to several tasks that are prioritized according to the value of the tasks
	Monarchic	One prefers to work on tasks that allow complete focus on one thing at a time
	Oligarchic	One prefers to work on multiple tasks in service of multiple objectives without setting priorities
	Anarchic	One prefers to work on tasks that allow flexibility as to what, where, when, and how one works
Level	Global	One prefers to pay more attention to the overall picture of an issue and to abstract ideas
	Local	One prefers to work on tasks that require working with concrete details
Scope	Internal	One prefers to work on tasks that allow one to work as an independent unit
	External	One prefers to work on tasks that allow for collaborative ventures with other people
Leaning	Liberal	One prefers to work on tasks that involve novelty and ambiguity
	Conservative	One prefers to work on tasks that allow one to adhere to the existing rules and procedures when performing tasks

**Table 3:** Comparison of differences in classroom environments across school types.

	TR	PR	TM	TA	CA	TE	PE
School Type	M ± SD	M ± SD	M ± SD	M ± SD	M ± SD	M ± SD	M ± SD
A	3.26 ± 0.69	3.07 ± 0.41	3.48 ± 0.57	4.05 ± 0.66	3.09 ± 0.55	3.93 ± 0.68	3.24 ± 0.48
B	3.24 ± 0.66	3.27 ± 0.59	3.30 ± 0.55	3.95 ± 0.62	2.99 ± 0.50	3.25 ± 0.50	3.14 ± 0.54
C	3.00 ± 0.62	3.14 ± 0.52	3.26 ± 0.52	3.92 ± 0.73	2.09 ± 0.45	3.05 ± 0.48	3.02 ± 0.49
F	7.00 ***	2.23 *	7.02 ***	5.58 *	11.12 ***	3.06 *	7.47 ***

Note: TR: teacher-student relationship; PR: peer relationship; TM: teaching methods; TA: teachers’ attitude; CA: classroom atmosphere; TE: teachers’ evaluation; PE: physical environment. Note: A: city-level schools; B: county-level schools; C: rural-level schools. *** *p* < 0.001, ** *p* < 0.01, * *p* < 0.05.

**Table 4:** Means and SD of classroom environment, thinking style, and Chinese language creativity.

Variable	*M*	*SD*	Variable	*M*	*SD*
**Thinking style**			**Classroom environment**		
**Function**			Teacher-student relationship	3.60	0.82
Legislative style	4.87	1.02	Peer relationship	3.46	0.55
Executive style	4.65	1.06	Teaching methods	3.62	0.59
Judicial style	4.51	1.09	Teachers’ attitudes	3.98	0.67
**Form**			Classroom environment	3.55	0.66
Monarchic style	3.76	1.19	Teacher’s evaluation	3.39	0.56
Hierarchical style	4.80	1.23	Physical environment	3.25	0.51
Oligarchic style	3.93	1.22	**Chinese language creativity**		
Anarchic style	4.47	1.09	Fluency	6.33	3.01
**Level**			Flexibility	6.06	1.88
Global style	3.77	1.19	Originality	1.58	0.85
Local style	4.38	0.96			
**Scope**					
Internal style	4.00	1.08			
External style	4.92	1.10			
**Learning**					
Liberal style	4.64	1.00			
Conservative style	4.17	0.99			

**Table 5:** Comparison of thinking style differences between high- and low-language creativity groups.

Variable		High Language Creativity		Low Language Creativity		
Thinking Style		*M*	*SD*	*M*	*SD*	*t*
Function	Legislative style	5.04	1.01	4.70	1.02	2.60 **
	Executive style	4.72	1.12	4.35	1.08	2.62 **
	Judicial style	4.68	1.11	4.32	1.07	2.58 **
Form	Monarchic style	3.56	1.14	3.75	1.15	1.25
	Hierarchical style	4.94	1.22	4.60	1.29	2.11 *
	Oligarchic style	3.78	1.34	3.94	1.17	0.97
	Anarchic style	4.61	0.99	4.33	1.45	2.10 *
Level	Global style	3.74	1.20	3.88	1.20	0.90
	Local style	4.47	1.00	4.21	1.01	2.07 *
Scope	Internal style	4.14	1.14	3.89	1.07	1.77
	External style	4.96	1.07	4.85	1.21	0.75
Learning	Liberal style	4.83	1.02	4.49	0.98	2.62 **
	Conservative style	4.17	1.02	4.09	1.03	0.60

Note: * *p* < 0.05, ** *p* < 0.01.

**Table 6:** Moderate regression results in language creativity.

Step Sand Variables	Fluency			Flexibility			Originality		
	Model 1	Model 2	Model 3	Model 1	Model 2	Model 3	Model 1	Model 2	Model 3
*R* ^2^	0.045	0.085	0.373	0.027	0.084	0.320	0.022	0.056	0.304
Adjust *R*^2^	0.030	0.042	0.168	0.011	0.042	0.097	0.007	0.012	0.077
△*R*^2^	0.045	0.040	0.288	0.027	0.057	0.236	0.022	0.034	0.248
△*F*	2.969	1.441	1.714	1.744	2.073	1.290	1.429	1.192	1.329
Sig. *F* Change	0.005	0.137	0.000	0.097	0.015	0.056	0.192	0.282	0.038

**Table 7:** Classroom Environment and Thinking Style Effects on Fluency and Originality.

Thinking Style	Fluency		Originality	
	*β*	*t*	*β*	*t*
(Constant)	6.583	42.461 ***	1.524	33.131 ***
*Z* (Teacher-student relationship)	0.245	1.041	−0.031	−0.45
*Z* (Peer relationship)	−0.5	−2.492 *	−0.188	−3.159 **
*Z* (Teaching methods)	0.195	1.023	0.132	2.327 *
*Z* (Teachers’ attitude)	−0.189	−0.903	−0.028	−0.448
*Z* (Classroom atmosphere)	−0.104	−0.504	0.004	0.063
*Z* (Teachers’ evaluation)	0.653	3.671 ***	0.084	1.585
*Z* (Physical environment)	0.173	0.937	−0.029	−0.521
*Z* (Legislative style)	0.38	1.874	0.044	0.731
*Z* (Executive style)	0.122	0.645	0.096	1.721
*Z* (Judicial style)	0.064	0.321	−0.079	−1.35
*Z* (Monarchic style)	−0.096	−0.54	0.013	0.241
*Z* (Hierarchical style)	−0.13	−0.611	0.077	1.214
*Z* (Oligarchic style)	−0.017	−0.089	−0.01	−0.178
*Z* (Anarchic style)	0.409	1.991 *	0.05	0.829
*Z* (Global style)	0.088	0.471	−0.016	−0.282
*Z* (Local style)	0.062	0.314	0.013	0.22
*Z* (Internal style)	−0.153	−0.814	0.001	0.014
*Z* (External style)	−0.587	−2.878 **	−0.056	−0.918
*Z* (Liberal style)	0.186	0.896	0.058	0.936
*Z* (Conservative style)	−0.084	−0.416	−0.076	−1.227

Note: *** *p* < 0.001, ** *p* < 0.01, * *p* < 0.05.

**Table 8:** Coefficients of equations with interacting items of fluency.

Thinking Style		Fluency	
		*β*	*t*
Function	Teachers’ attitude * Legislative	−0.605	−2.020 *
	Classroom atmosphere * Executive	0.623	2.568 *
	Classroom atmosphere * Anarchic	0.7	2.469 *
	Teachers’ evaluation * Anarchic	−0.668	−2.951 *
	Teaching methods * Anarchic	0.703	3.092 **
	Teachers’ attitude * Anarchic	−0.701	−2.432 *
	Teacher-student relationship * Local	−0.568	−2.054 *
	Physical environment * Global	−0.565	−2.344 *
Scope	Teachers’ attitude * Internal	0.978	3.369 **
	Teachers’ evaluation * Internal	−0.533	−2.277 *
	Peer relationship * Internal	−0.528	−2.286 *

Note: *** *p* < 0.001, ** *p* < 0.01, * *p* < 0.05.

**Table 9:** Coefficients of equations with interacting items of originality.

Thinking Style		Originality	
		*β*	*t*
Function	Teachers’ attitude * Executive	0.195	2.290 *
Form	Peer relationship * Hierarchical	−0.15	−2.027 *
	Teachers’ evaluation * Hierarchical	0.19	2.647 **
Level	Peer relationship * Local	0.189	2.503 *
	Teachers’ evaluation * Local	−0.16	−2.087 *
	Classroom atmosphere *Internal	0.151	2.013 *
	Teacher-student relationship * Internal	0.193	2.061 *
	Physical environment * External	−0.197	−2.392 *

Note: *** *p* < 0.001, ** *p* < 0.01, * *p* < 0.05.

**Table 10:** The match-or-not of a high preference for one’s thinking style and the high quality of the classroom environment and its effect on students’ creativity.

Classroom Environment * Thinking Style	Meaning of Classroom Environment	Meaning of Thinking Style	Match or Not	Effect
Teachers’ attitude * Legislative	Teachers respect students, are more tolerated, and are friendly	One prefers tasks requiring creative strategies and autonomy	Match	Inhibited fluency
Teachers’ attitude * Executive		One prefers tasks with clear instructions and established guidelines.	Not match	Enhance originality
Classroom atmosphere * Executive	The class is more open-minded, creative, encouraging, and has a high tolerance for different ideas		Not match	Enhanced fluency
Classroom atmosphere * Judicial		One prefers tasks involving evaluation and judgment of others.	Not match	Inhibited fluency
Peer relationship * Judicial	Students hold a reliable, united, appropriate, competitive, encouraging, and respectful relationship		Not match	Enhanced fluency
Peer relationship * Monarchic		One prefers tasks that allow focused work on a single aspect.	Not match	Inhibited originality
Peer relationship * Hierarchical		One prefers to allocate attention to several tasks that are prioritized according to their value	Not match	Inhibited originality
Teachers’ evaluation * Hierarchical	Teachers’ evaluation tends to be more positive and encouraging		Match	Enhanced originality
Teachers’ evaluation * Anarchic		One prefers to work on tasks that allow flexibility as to what, where, when, and how one works	Match	No difference
Classroom atmosphere * Anarchic	The class is more open-minded, creative, encouraging, and has a high tolerance for different ideas		Match	Enhanced fluency
Teaching methods * Anarchic	The teaching style is more open, variable, and creative		Match	Enhanced fluency
Teachers’ attitude * Anarchic	Teachers respect students, are more tolerated, and are friendly		Match	Inhibited fluency
Peer relationship * Local	Students hold a reliable, united, appropriate, competitive, encouraging, and respectful relationship.	One prefers to work on tasks that require working with concrete details		No difference
Teachers’ evaluation * Local	Teachers’ evaluations tend to be more positive and encouraging			Inhibited originality
Teacher–student relationship * Local	The relationship is more democratic; teachers and students have equal status; they both contribute to students” development; and they respect each other			Inhibited fluency
Physical environment * Global	A higher score means a more creative-friendly physical environment, like desks that can be arranged according to specific themes in class, well-lit bulbs, and reduced noise	One prefers to pay more attention to the overall picture of an issue and to abstract ideas		Inhibited fluency
Teachers’ attitude * Internal	Teachers respect students, are more tolerated, and are friendly	One prefers to work on tasks that allow one to work as an independent unit	Not match	Inhibited fluency
Teachers’ evaluation * Internal	Teachers’ evaluations tend to be more positive and encouraging		Not match	Enhanced fluency
Classroom atmosphere * Internal	The class is more open-minded, creative, encouraging, and has a high tolerance for different ideas		Not match	Enhanced originality
Peer relationship * Internal	Students hold a reliable, united, appropriate, competitive, encouraging, and respectful relationship		Not match	Inhibited fluency
Teacher–student relationship * Internal	The relationship is more democratic; teachers and students have equal status; they both contribute to students” development; and they respect each other		Not match	Enhanced originality
Physical environment * External	A higher score means a more creative-friendly physical environment, like desks that can be arranged according to specific themes in class, well-lit bulbs, and reduced noise.	One prefers to work on tasks that allow for collaborative ventures with other people	Match	Inhibited originality

Note: * denotes the interaction effect between the variables.

## Data Availability

Data are protected due to student privacy; for access to the data, please contact the corresponding author.
